# Hypoglycemia identified by a continuous glucose monitoring system in a second-trimester pregnant woman with insulinoma: a case report

**DOI:** 10.1186/s13256-017-1265-8

**Published:** 2017-04-21

**Authors:** Marjeta Tomazic, Andrej Janez, Maja Ravnik Oblak

**Affiliations:** Department of Endocrinology, Diabetes and Metabolic Diseases, University Medical Center Ljubljana, Zaloška 7, Ljubljana, 1525 Slovenia

**Keywords:** Insulinoma, Pregnancy, Hypoglycemia, Continuous glucose monitoring system

## Abstract

**Background:**

Insulinoma associated with pregnancy is a very rare condition and can be difficult to diagnose. Here, we present an interesting case of insulinoma occurring during pregnancy with special attention paid to the use of a continuous glucose monitoring system to detect hypoglycemia.

**Case presentation:**

A 36-year-old white woman in the second trimester of pregnancy presented with recurrent episodes of hypoglycemia associated with neuroglycopenic symptoms. The use of a continuous glucose monitoring system confirmed hypoglycemia. Serum insulin, C-peptide, and proinsulin values confirmed endogenous hyperinsulinism. A tumor mass was localized at the tail of her pancreas by endoscopic ultrasound and confirmed by magnetic resonance imaging. Surgery performed at 21 weeks of gestation by distal pancreatectomy confirmed the presence of a 15 mm diameter endocrine tumor at the tail of her pancreas and led to a cure.

**Conclusions:**

Hypoglycemia during pregnancy could be due to insulinoma. Use of a continuous glucose monitoring system could help to detect hypoglycemia in these patients.

## Background

Insulinoma is the most common neuroendocrine tumor of the pancreas and causes hypoglycemia due to endogenous hyperinsulinism. Confirmation of diagnosis is usually obtained by showing an association of hypoglycemia with non-suppressed insulin secretion during normal or prolonged fasting. It has an estimated incidence of one to four cases per million per year, based on population study [1]. The incidence has been reported to be higher in autopsy studies (0.8 to 10%), suggesting that these tumors frequently remain undiagnosed [2]. The median age of patients at presentation is approximately 47 years, and females show a slight predominance over males [3].

It is very common for some patients to develop hypoglycemic unawareness as a result of central nervous system adaptation to chronic hypoglycemia, and severe neuroglycopenic symptoms such as confusion and loss of consciousness with seizures and coma can occur [4]. With the introduction of new highly specific insulin assays, lower levels of insulin have been detected in patients with insulinomas; thus, new lower diagnostic criteria for the diagnosis of inappropriate hyperinsulinemia in the presence of documented hypoglycemia proposed a diagnostic threshold of plasma insulin of 3 mU/l [5].

Insulinoma associated with pregnancy is a very rare condition and can be difficult to diagnose. Approximately 30 cases of insulinoma presenting in pregnancy have been reported and its management is very challenging. In most cases, hypoglycemia occurred during the first trimester or within 2 weeks after delivery [6, 7].

It is well established that clinical signs and symptoms of insulinoma can be attenuated and in some cases can even be masked by pregnancy. Furthermore, such a possibility has to be taken into account in the differential diagnosis of symptomatic hypoglycemia during the first trimester of pregnancy or in the postpartum period. Therefore, in the present case report of insulinoma occurring during pregnancy, special attention is paid to the use of a continuous glucose monitoring system (CGMS) to detect hypoglycemia.

## Case presentation

A 36-year-old white woman, 73 kg, 1.64 m, body mass index (BMI) 27.1 kg/m2, gravida 1 in 17 weeks of gestation presented to our hospital for the first time for evaluation of recurrent episodes of hypoglycemia associated with neuroglycopenic symptoms. She had no chronic illness or medical conditions and had not taken any medication prior to hospital admission. Her family and social history were not remarkable. She had post-high-school education and was not employed. Her environmental history revealed no abnormalities. Neither parent smoked tobacco during the pregnancy or took any drugs or medication. In 2008, she had a normal pregnancy with no complications and gave birth to a healthy 3500 g baby boy. On physical examination her vital signs were normal, her blood pressure was 125/75 mmHg, and pulse 85/minute. The examination was otherwise unremarkable. Her neurological examination was normal. An initial blood test of complete blood count (CBC), liver test, and renal function as well as urine analysis were in normal laboratory range.

To detect and evaluate her episodes of spontaneous hypoglycemia a CGMS was administered for 3 days. The system used was the MiniMed® (Medtronic, Northridge, CA, USA) subcutaneous CGMS® System™. This system continuously measures subcutaneous tissue interstitial glucose levels, recording values on average every 5 minutes within a range of 2.2 to 22.0 mmol/L. It allows 288 measurements of glucose in a 24-hour period.

**Fig. 1:**
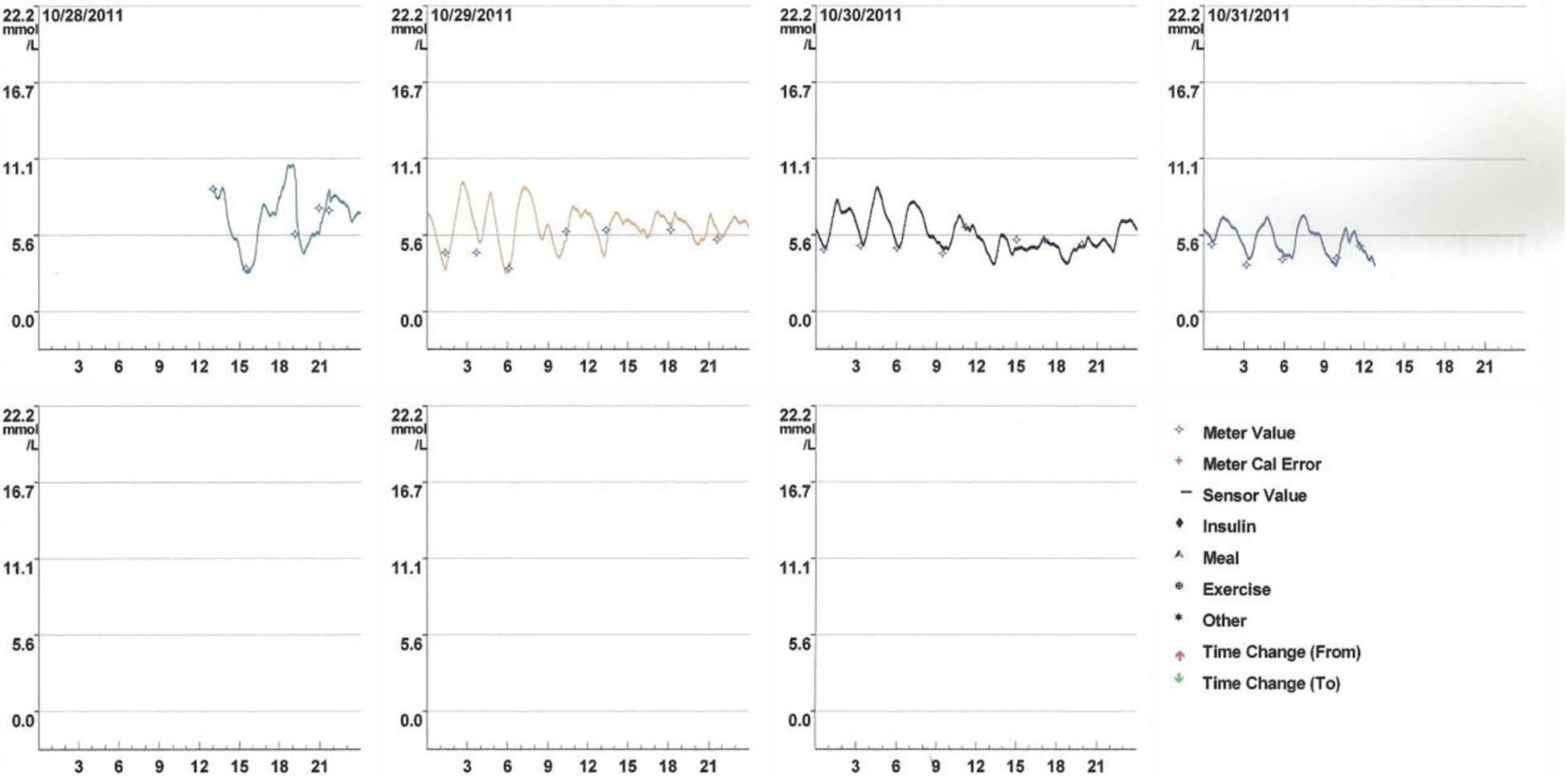
Continuous glucose monitoring system revealed the hypoglycemic episodes

The CGMS (Fig. 1) detected hypoglycemia 3 hours after her last ingestion of food. Her interstitial glucose value was 3.1 mmol/l, confirmed by plasma glucose value of 2.7 mmol/l, serum insulin 4.25 mE/l, C-peptide 0.54 nmol/l, and proinsulin value of 7.49 ulU/ml. After correction with a carbohydrate meal, hypoglycemia was again confirmed the next day at 1 a.m. with an interstitial glucose value of 3.3 mmol/l, a serum glucose value of 2.9 mmol/l, serum insulin 6.3 mE/l, C-peptide 0.44 nmol/l, and proinsulin 13.70 pmol/l, clearly indicating endogenous hyperinsulinism. Another instance of hypoglycemia was confirmed at 9 a.m. An adjusted meal plan with a proper amount of carbohydrate did not cause any further hypoglycemia as observed by the CGMS.

**Fig. 2:**
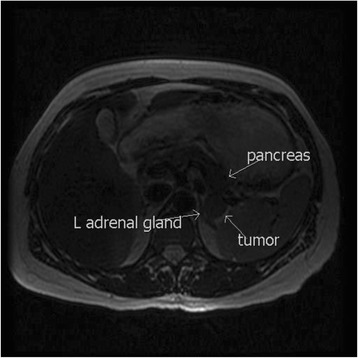
Magnetic resonance imaging demonstrated hyperintense solid lesion 2 cm in diameter localized in the area of the pancreatic tail

Preoperatively, a tumor mass of 18×22 mm was localized at the tail of her pancreas by endoscopic ultrasound. Magnetic resonance imaging (MRI) was subsequently performed and no contrast medium was used because of the pregnancy. MRI demonstrated a hyperintense solid lesion 2 cm in diameter localized in the area of her pancreatic tail (Fig. 2). Surgical exploration at 21 weeks of gestation confirmed one tumor in her pancreatic tail near to lienal artery and distal pancreatectomy and splenectomy was performed. A histopathological examination confirmed a 15 mm, partly encapsulated and partly expansive tumor with strong immunohistochemical staining for chromogranin and focally for insulin. There were also signs of vascular or perineural invasion. Tumor cells showed mild atypias with nucleolar enlargement, a slightly increased nuclear/cytoplasmic ratio, and nuclear crowding. Ki-67 staining indicated a proliferative index of approximately 5%, which is diagnostic for a low-grade malignancy. On macroscopic examination, there were no signs of metastases and pT3N0Mx tumor staging was determined.

After surgical excision of the tumor, our patient had no more hypoglycemic attacks. Her postoperative blood glucose levels were within the normal range. Her serum insulin and C-peptide levels after oral glucose tolerance test (OGTT) were in the normal range

**Fig. 3:**
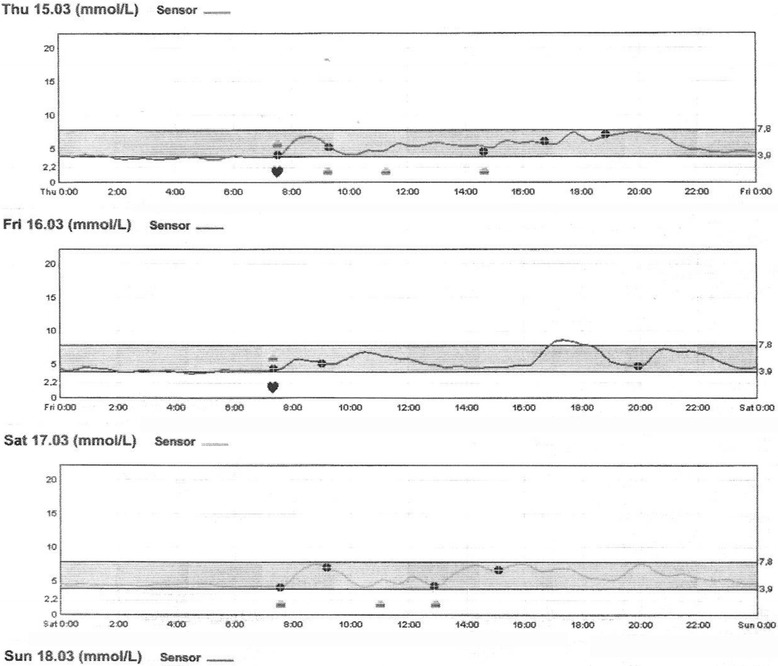
A 72-hour continuous glucose monitoring system was used as a supplementary tool for glucose control evaluation after she was diagnosed with gestational diabetes

The pregnancy continued without complications; at week 28 she was diagnosed with gestational diabetes. She was successfully treated with diet (Fig. 3). During the pregnancy she gained 10 kg of weight. She delivered vaginally after 39 weeks of gestation: a baby girl with an Apgar score of 9/10 at 1 and 5 minutes. A birth weight of 3.57 kg, head circumference of 37 cm, and length at birth of 50 cm were recorded. No dyschromias or malformations were found. The examination of the newborn was normal. Her blood glucose level was normal. The postpartum period was without complication for the baby and for the mother. She was breastfed until the age of 9 weeks. She had regular visits to our diabetology out-patient clinic every 3 months. Her glucose levels and glycated hemoglobin (HbA1c) were in normal range. She is without signs of recurrence or metastatic disease 36 months after tumor removal. Her glucose tolerance, checked by OGTT, is normal.

## Discussion

The classical diagnosis of insulinoma depends on confirming the criteria of Whipple’s triad: symptoms, signs, or both consistent with hypoglycemia, a low plasma glucose concentration, and resolution of those symptoms or signs after the plasma glucose concentration is raised [6]. In adults with symptoms of neuroglycopenia or documented low blood glucose levels, the gold standard for biochemical diagnosis remains measurement of plasma glucose, insulin, C-peptide, and proinsulin during a 72-hour fast [8, 9].

The diagnosis of insulinoma is often not suspected during the first or at the beginning of the second trimester of pregnancy because weakness, nausea, hypotension, and even mild episodes of hypoglycemia are common at the beginning of normal pregnancy [7].

The signs and symptoms occur at a time of fasting and include weakness, dizziness, confusion, personality changes, and loss of consciousness, seizures, and coma. Many pregnant patients with insulinoma are misdiagnosed and treated with anticonvulsant therapy. Because of the often confusing clinical picture of pregnant women with insulinoma, a possibility of hypoglycemia has to be taken into account in the differential diagnosis of personality and consciousness changes during the first trimester of pregnancy or in the postpartum period.

Particular adaptation in insulin sensitivity during pregnancy could explain why most of the previous reported cases showed suggestive hypoglycemic signs by week 16 of gestation, before insulin resistance appeared. Only one of the patients with malignant insulinoma in the report became symptomatic by week 35. This might be explained due to different insulin sensitivity in the course of pregnancy. Frequent meals and intravenously administered glucose infusions were adequate for hypoglycemia control in most of the cases, and only a small number of patients were operated at weeks 12 to 17 of gestation [10].

Late in pregnancy, with the rise in insulin resistance, clinical hypoglycemia can be absent or attenuated, so the tumors may be masked. Hypoglycemic episodes may be absent during the second half of pregnancy even in the presence of a confirmed insulinoma, but in the postpartum period they often recur after a rebound in insulin sensitivity [7].

To assess glucose concentrations more frequently, CGMSs were developed for patients with diabetes almost 2 decades ago. Their use might also be useful to detect glucose fluctuations and specially hypoglycemia in patients with insulinoma. The system measures glucose concentrations in the subcutaneous interstitial fluid, which has been shown to correlate well with that obtained in whole blood. In humans, the average delay between a change in the glucose concentration in blood versus interstitial fluid was approximately 5 minutes, which makes the CGMS reliable for real-time monitoring [11]. Hypoglycemia needs to be treated promptly. The American Diabetes Association recommends treatment with 15 to 20 grams of carbohydrate. In order to treat early symptoms of hypoglycemia, patients should be certain that fast-acting carbohydrate (such as glucose tablets, 1 cup of nonfat milk, 1 tablespoon of sugar, or sweetened fruit juice) is available at all times. Fifteen to 20 grams is usually sufficient to raise blood glucose into a normoglycemic range without inducing hyperglycemia. This can be followed by long-acting carbohydrate to prevent recurrent symptoms. However, in the case of severe hypoglycemia, when a woman is unable to eat or drink something, emergency medical attention is required. Alternatively, a glucagon kit for home use should be prescribed [12]. In our patient a proper carbohydrate meal and the use of CGMS prevented further hypoglycemia. To the best of our knowledge, this is the first case report of insulinoma during pregnancy where CGMS could help to detect and avoid hypoglycemia. It is well established that CGMS allows identification of patterns of glucose levels for up to 72 hours with details of excursions above and below target levels and, thus, may be a valuable adjunct tool in hypoglycemia detection and prevention in this condition. In our patient we used the Medtronic MiniMed CGMS and uncovered a significant rate of hypoglycemia that previously might not have been detected by conventional means. Although repeated hypoglycemia has caused teratogenic effects in animal models, no fetal malformation has been described in previous reports of insulinoma during pregnancy, whether cured or not [9]. This is in agreement with prospective studies in insulin-treated pregnant women with diabetes showing no correlation between hypoglycemia and malformations. These results are encouraging with respect to such pregnancies which, however, require careful supervision.

The most reliable confirmatory test is prolonged fast. It is well established that pregnancy does not alter the ability of hypoglycemia to suppress insulin secretion; therefore, the diagnosis of endogenous hyperinsulinism can be made by prolonged fasting as in the non-pregnant state [8]. In a pregnant woman, this test has to be done under very close supervision because pregnant women can have a much more pronounced response to hypoglycemia. A high index of suspicion is required for the diagnosis since abnormal immunoreactive insulin and glucose ratios greater than 0.3 can be found in non-insulinoma normal pregnancy due to amplified islet cell responsiveness [7, 8].

If diagnosis is confirmed during gestation, surgery can be performed in the second trimester. In some cases, a conservative approach might be advised until delivery, to proceed to definitive surgical treatment preceded by tumor localization, after the end of pregnancy [10, 13]. In our case, surgical exploration at 21 weeks of gestation confirmed the preoperative localization and the tumor was completely removed by distal pancreatectomy. Deciding to conduct surgery before the end of pregnancy seemed reasonable because the patient needed constant glucose infusion to prevent hypoglycemia. Furthermore, preoperative morphological diagnostics suggested the invasiveness of the tumor and postoperative histological examination showed low mitogenic potential.

## Conclusions

Insulinoma complicating pregnancy, although very rare, should now be included in the differential diagnosis of hypoglycemia during pregnancy. Use of a CGMS could help to detect hypoglycemia in these patients.

## Abbreviations

CGMS: Continuous glucose monitoring system
MRI: Magnetic resonance imaging
OGTT: Oral glucose tolerance test
